# An easy and reproducible method for a large-zone deep partial-thickness burn model in the mini-pig

**DOI:** 10.1093/burnst/tkae086

**Published:** 2025-02-17

**Authors:** Ugo Lancien, Maria Sbeih, Alexandra Poinas, Pierre Perrot, Selim Aractingi, Amir Khammari, Brigitte Dréno

**Affiliations:** Plastic, Reconstructive and Aesthetic Surgery Unit, Burn Unit for Adults and Children, Hôtel Dieu, University Hospital Nantes, 1 Place Alexis Ricordeau, Nantes F-44000, France; INSERM, UMRS 1229, Laboratory Regenerative Medicine and Skeleton (RMeS), 1, Place Alexis Ricordeau, Nantes F44000, France; Cutaneous Biology Lab, Institut Cochin, INSERM U1016, UMR 8104, 24 rue du Faubourg St Jacques, Paris 75014, France; Clinical Investigation Centre CIC1413, CHU Nantes and INSERM, 1 Place Alexis Ricordeau, Nantes F-44000, France; Plastic, Reconstructive and Aesthetic Surgery Unit, Burn Unit for Adults and Children, Hôtel Dieu, University Hospital Nantes, 1 Place Alexis Ricordeau, Nantes F-44000, France; Department of Dermatology, Université Paris 5 Descartes, ApHp and Inserm Institut Cochin 2f016, Hôpital Cochin, 27 Rue du Faubourg Saint-Jacques, Paris F-75014, France; Department of Dermatology, Nantes University, CIC 1413, INSERM UMR 1302/EMR6001 INCIT, CHU de Nantes, 1 Place Alexis Ricordeau, Nantes F-44000, France; Nantes Université, INSERM, CNRS, Immunology and New Concepts in ImmunoTherapy, INCIT, UMR 1302/EMR6001, Nantes F-44000, France

Partial-thickness dermal burn (PTDB) causes devastating trauma to the skin, resulting in an increased risk of infections and scars [1]. PTDB studies allow to better understand its pathophysiology and to improve the management of PDTB lesions [2].

*In vivo* animal burn models help to study the epithelialization process, contraction, and scar formation, as well as to test the effectiveness of therapies that support the wound healing process [3–5]. The porcine model may be considered the most suitable *in vivo* deep burn model, as pig skin resembles the most to that of humans [3]. However, easily reproducible PTDB models in the pig do not yet exist.

The present two *in vivo* mini-pig studies aimed to develop an easy-to-use and reproducible PTDB model.

Studies were conducted, in accordance with local regulations and received approval from the local ethics committee in June 2022.

Four mini-pigs (*Sus scrofa domesticus*, Yucatan), aged 18 months were purchased from INRAE (*Institut national de Recherche pour l’Agriculture, l’alimentation et l’Environnement*, UMR1348 PEGASE, Saint-Gilles, France). Animals received standard diet and water *ad libitum* and were left to adapt to the conditions for a week before the procedure. Animals fasted the night before the procedure. Individual housing was performed during the experimental phase.

All anesthesia and pain management procedures were conducted in accordance with the European Directive 2010/63/EU to ensure high standards of animal welfare.

The animals’ health status was monitored twice a day, and any sign of pain, wound infection, or sepsis was identified.

Euthanasia was conducted by overdosing with intravenous (IV) 140 mg/ml pentobarbital (Dolethal®, Vetoquinol, France).

The paravertebral region was chosen for large burn lesions [4].

During the first study, a glass bottle (Schott, Germany) and a locally manufactured brass pestle (rectangular shape; 8 × 5 × 5cm, 1.7 kg; Figure S1, see online supplementary file) were tested. The glass bottle procedure was described previously [5,6]. The pestle procedure was inspired by a method presented by Seswandhana *et al*. and adapted to the present model [4].

Both devices were heated in a hot water bath or an oven. Heated devices were applied firmly on predefined zones (8 × 5 cm) of the animals’ dorsal skin (Figure S2, see online supplementary file). The temperature was controlled using a contact thermometer immediately before application on each zone.

The burned skin was removed using gauze moistened with a physiological solution. Loss of epidermis, blister formation, visualization of whitened dermis, and delay or loss of capillary refill when pressured were observed. Animals were sacrificed at the end of the study.

A second study was conducted on one mini-pig using the device that provided the desired PTDB lesions. Burns were performed on three predefined and identified zones on the animal’s dorsal skin. Biopsy samples were collected at D3, D14, and D28 using biopsy punches of 8 mm (Paramount, Ohkla, India) from the border of each wound, including burned and healthy skin equally (Figure S3 see online supplementary file). Wound dressings were applied following a protocol specifically developed for this study (Figure S4, see online supplementary file).

Skin samples were fixed with paraformaldehyde 4% before being embedded in cryomolds with a freezing medium, then flash-frozen in isopentane at −80°C. Biopsies were cut in longitudinal sections of 10 μm using a cryostat (NX 70®, Thermo Fisher, MM France) and stained with haematoxylin–eosin (HE) or using anti-Keratin (K) 14 labelling. Photos were taken using a Lamina® slide scanner (Akoya Perkin Elmer, California, USA). The animal was sacrificed after 28 days.

According to the macroscopic evaluation, burns realized with the glass bottle were nonhomogeneous across the zone and did not meet the macroscopic criteria of a deep partial thickness burn at all tested temperatures (60°C, 92°C, 95°C, and 100°C) and using both heating methods.

Burns realized with the 60°C-heated glass were superficial with a red, dry, and smooth skin; without blisters; and with a normal capillary refill. Burns realized at 92°C, 95°C, or 100°C using the bottle were of more superficial partial-thickness severity, with a normal capillary refill and a blistering and destroyed epidermis, exposing an underlying red skin.

Conversely, burns realized with the brass pestle were homogeneous. The 95°C- and 100°C-heated brass pestle provided PTDB at all application times. The skin was necrotic, brown, hard, dry, and without blisters, indicating complete destruction of the epidermis and dermis. The 92°C-heated brass pestle applied firmly for 30 s provided macroscopically satisfying PTDB, meeting the fixed criteria (Figure 1). Capillary refill was absent, and the epidermis was destroyed with blisters uncovering a white dermis, indicating that deep layers of the dermis were reached.

**Figure 1:**
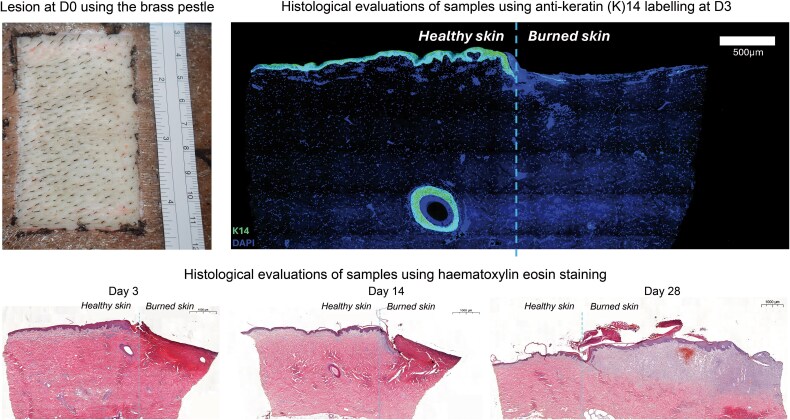
Visual lesion evaluation at D0 postburn using the brass pestle and histological examination at D3, 14, and 28 (Study B)

The brass pestle heated at 92°C in an oven and firmly pressed on the skin for 30 s was used for the second study.

Histology evaluations on HE- and K14-stained samples from D3 showed complete destruction of the epidermis compared to the surrounding healthy skin. The dermal tissue was eosinophilic and homogenized, with loss of normal cellular details and fibrillar architecture indicating the denaturation of collagen proteins with a loss of their triple-helix design, causing disorganized collagen fibres. Widened spaces between some dermal fibres indicated interstitial fluid accumulation or oedema. The damage in the dermis extended through the papillary dermis, reaching the middle section of the reticular dermis and leaving the underneath remaining reticular dermis intact with a normal cellular structure.

Most of the features observed at D3 persisted until D14. HE-stained samples revealed an eosinophilic and homogenized dermis extending from the papillary to the mid-reticular level, with widened spaces between dermal fibres. The formation of a neo-epidermis seemed to be initiated, along with the infiltration of immune cells in the subepidermal zone, as evidenced by the basophilic cluster observed under the neo-epidermis.

At D28, the neo-epidermis had covered a significant portion of the burned area and, being thickened, reflected the ongoing hyperproliferation of keratinocytes.

The reticular and papillary dermis was characterized by clearer cellular detail showing pronounced basophilia, indicating an ongoing collagen deposition and a high density of fibroblasts actively synthesizing collagen. The presence of red clusters suggested an increase in blood vessels and ongoing neovascularization, signaling a robust healing response. Figure 1 shows HE- and K14-stained biopsy samples of burned and intact skin at D3, D14, and D28.

Pictures taken at D28 showed a 49% re-epithelialization of the lesion, with several areas remaining unhealed (Figure S5, see online supplementary file).

We herewith propose an easy-to-implement and reproducible PTDB *in vivo* model in porcine skin, allowing to study the wound healing kinetics, tissue neocollagenesis, or the benefit of regenerative dressings.

In the past, different studies assessed the deep-burn potential of cylindrical brass blocks or rods [7]. However, in both cases, the authors obtained nonuniform superficial burns that were not suitable for deep-burn models. One reason why this method failed may reside in the delay between heating and applying the burning tool. In the present method, the temperature of the brass pestle was controlled immediately before its application, thus limiting a loss of temperature. The brass was also reheated using an oven before each application, allowing to reach the desired temperature in a more precise and faster way.

Wounds caused by burns at 92°C for 30 s using the brass pestle met the criteria for a partial deep-thickness dermal burn model. The 28-day re-epithelialization observation period allowed to follow up the healing process of these deep partial-thickness burn lesions. To our knowledge, this represents the longest postburn observation period reported in literature, contrasting with the typical 3- to 5-day healing observation period in studies involving primarily less severe burns [8].

Histological evaluations confirmed that the pestle used induced a partial-thickness burn with complete destruction of the epidermal layer, confirming a partial-thickness dermal burn lesion [9]. Although widened spaces observed between some dermal fibres may indicate oedema as described previously by Gibson *et al*. [10], this damage in the dermis extended through the papillary dermis, reaching the middle section of the reticular dermis and leaving intact areas of the reticular dermis that show a normal structure, thereby confirming the accuracy of the proposed PTDB lesion method [9].

Despite its easy-to-follow and reproducible design, the method has its limitations. In order to achieve PTDB lesions, it is important that the investigator who will perform the burn lesions follows a short training course to ensure that reproducible results will be obtained. Another limitation is the customized design and manufacture of the pestle. This presents both practical and financial challenges as variations in the material or thickness of the pestle may introduce biased results.

The proposed large-zone PTDB procedure, consisting of a rectangular brass pestle heated to 92°C in an oven and firmly applied for 30 s on the pig’s dorsal skin, is a highly reproducible burn method. It provides uniform deep partial-thickness burn lesions, enabling the monitoring of wound healing, re-epithelialization, and neo-collagenesis over 28 days.

## Supplementary Material

Suppl_Figure_1_tkae086

Suppl_Figure_2_tkae086

Suppl_Figure_3_tkae086

Suppl_Figure_4_tkae086

Suppl_Figures_for_rev_05022025_tkae086

## Data Availability

Data are available on request.

## References

[1] Chang S-J, Sartika D, Fan G-Y, Cherng JH, Wang YW. Animal models of burn wound management. In: Animal Models in Medicine and Biology; 2019. 10.5772/intechopen.89188.

[2] Guo HF, Ali RM, Hamid RA, Zaini AA, Khaza'ai H. A new model for studying deep partial-thickness burns in rats. Int J Burns Trauma. 2017;7:107–14. 29119063 PMC5665842

[3] Al-Deen Said S, Jatana S, Ponti AK, Johnson EE, Such KA, Zangara MT., et al. Development of a reproducible porcine model ofinfected burn wounds. J Biol Methods. 2022;9:e158. 10.14440/jbm.2022.379. PMC9058257 35510036

[4] Seswandhana R, Anzhari S, Ghozali A, Dachlan I, Wirohadidjojo YW, Aryandono T. A modified method to create a porcine deep dermal burn model. Ann Burns Fire Disasters. 2021;34:187–91. 34584509 PMC8396150

[5] Cuttle L, Kempf M, Phillips GE, Mill J, Hayes MT, Fraser JF., et al. A porcine deep dermal partial thickness burn model with hypertrophic scarring. Burns. 2006;32:806–20. 10.1016/j.burns.2006.02.023. 16884856

[6] Wang XQ, Kempf M, Liu PY, Cuttle L, Chang HE, Kravchuk O., et al. Conservative surgical debridement as a burn treatment: supporting evidence from a porcine burn model. Wound Repair Regen. 2008;16:774–83. 10.1111/j.1524-475X.2008.00428.x. 19128248

[7] Li J, Zhang YP, Zarei M, Zhu L, Sierra JO, Mertz PM., et al. A topical aqueous oxygen emulsion stimulates granulation tissue formation in a porcine second-degree burn wound. Burns. 2015;41:1049–57. 10.1016/j.burns.2014.11.016. 25554261

[8] Singer AJ, Berruti L, Thode HC, Jr, McClain SA. Standardized burn model using a multiparametric histologic analysis of burn depth. Acad Emerg Med. 2000;7:1–6. 10.1111/j.1553-2712.2000.tb01881.x. 10894235

[9] Chaudhary M, Bonde D, Patil S, Gawande M, Hande A, Jain D. Histopathological evaluation of tissue undergoing thermal insult. J Forensic Dent Sci. 2016;8:110. 10.4103/0975-1475.186361. PMC4970406 27555730

[10] Gibson ALF, Carney BC, Cuttle L, Andrews CJ, Kowalczewski CJ, Liu A., et al. Coming to consensus: what defines deep partial thickness burn injuries in porcine models? J Burn Care Res. 2021;42:98–109. 10.1093/jbcr/iraa132. 32835360 PMC7856457

