# Supplementary figures and images for: An easy and reproducible method for a large-zone deep partial-thickness burn model in the mini-pig
Source: Burns Trauma. 2025 Feb 17;13:tkae086. doi: 10.1093/burnst/tkae086 (PMC11831022; doi:10.1093/burnst/tkae086)

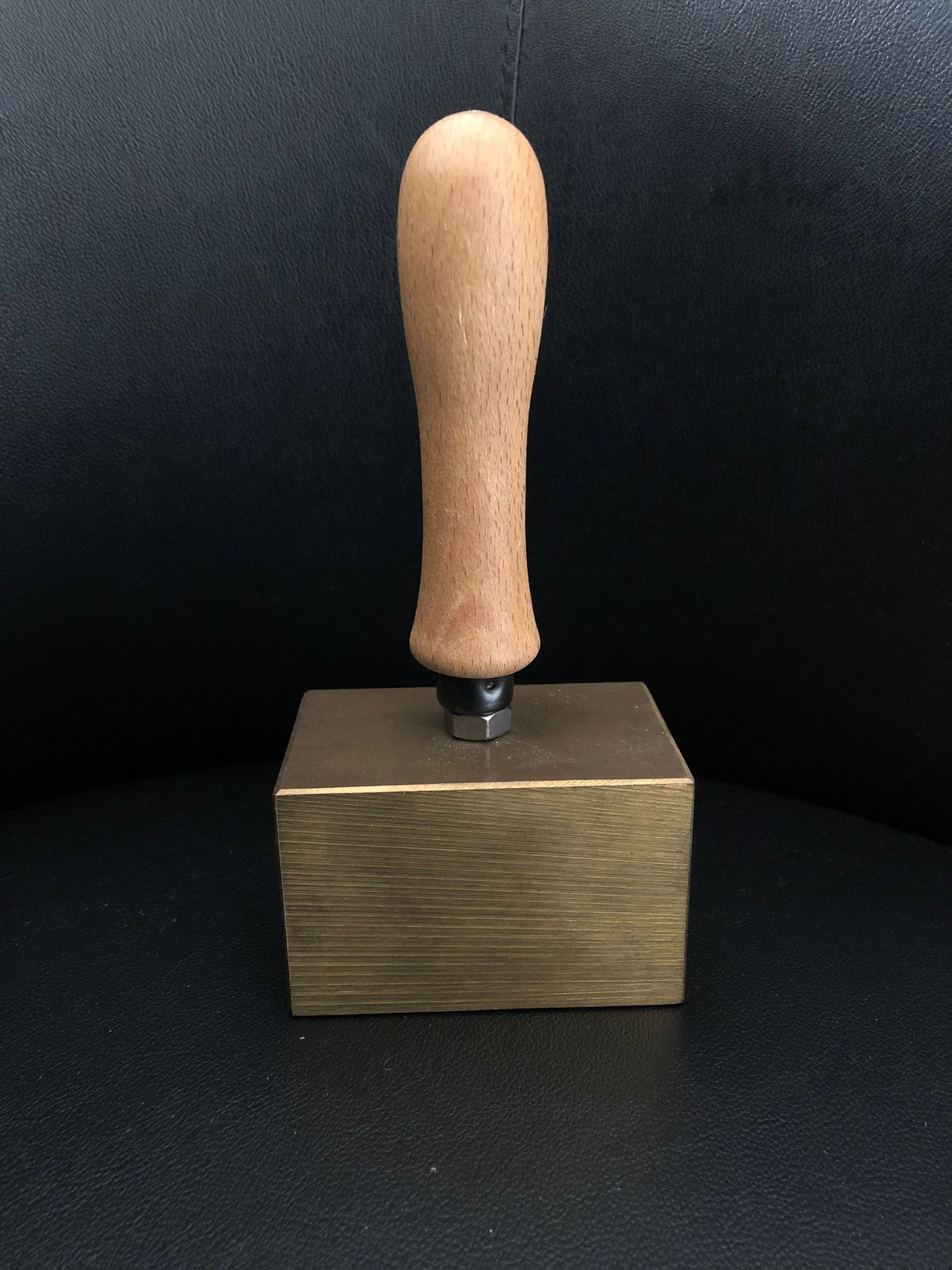

Supplement: Suppl_Figure_1_tkae086 [file suppl_figure_1_tkae086.jpeg]

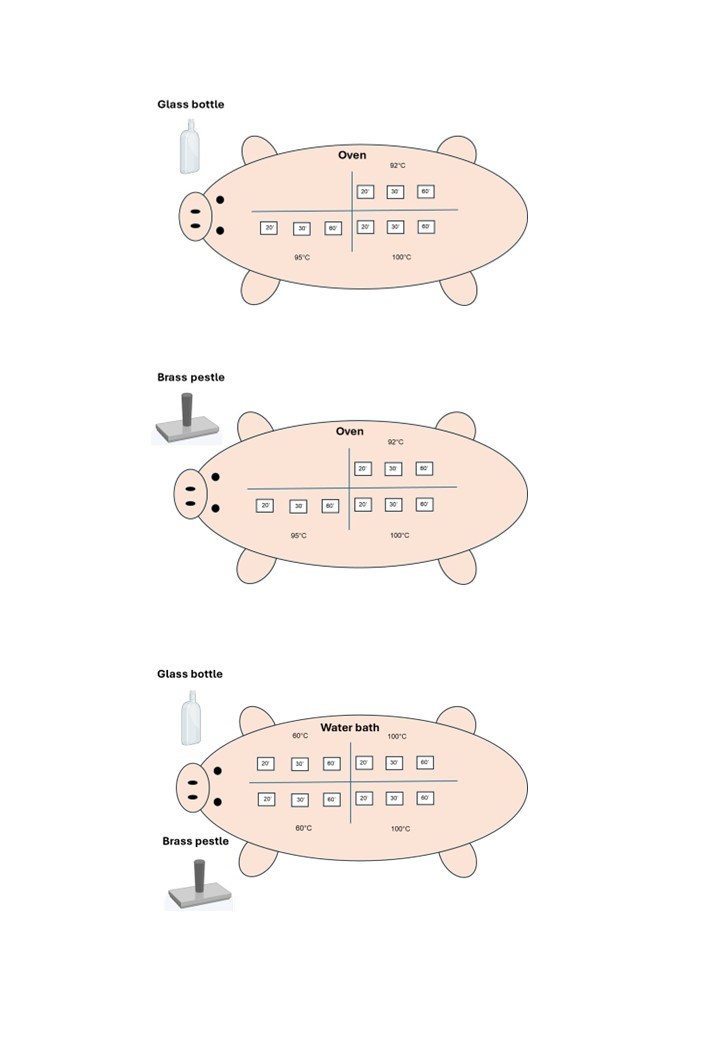

Supplement: Suppl_Figure_2_tkae086 [file suppl_figure_2_tkae086.jpeg]

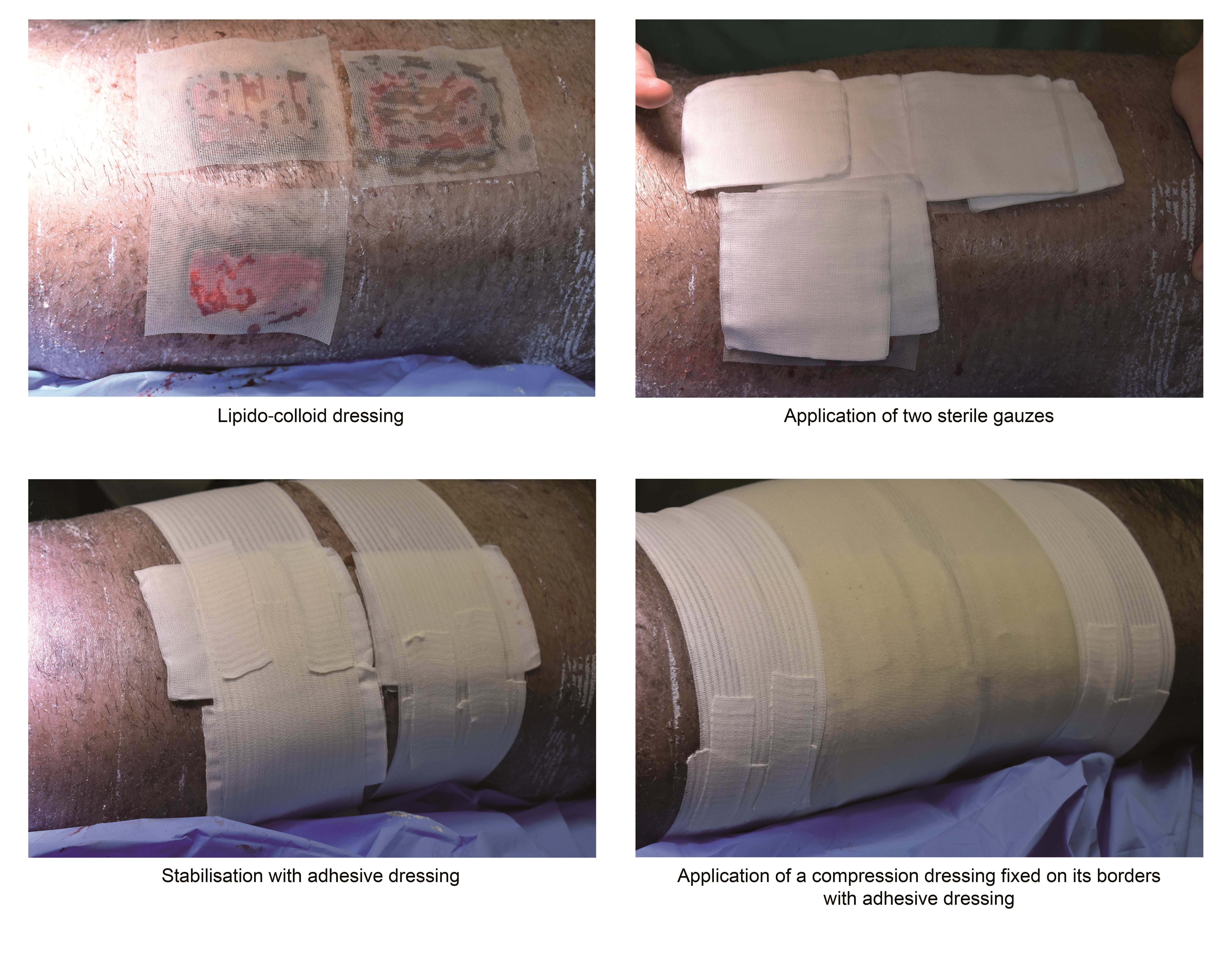

Supplement: Suppl_Figure_3_tkae086 [file suppl_figure_3_tkae086.jpeg]

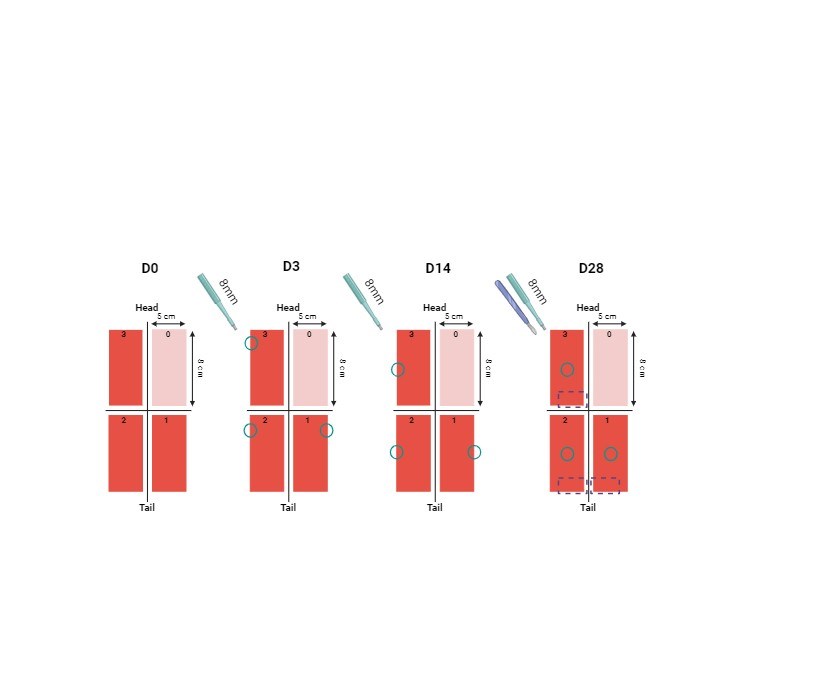

Supplement: Suppl_Figure_4_tkae086 [file suppl_figure_4_tkae086.jpeg]
