# Supplementary material for: An easy and reproducible method for a large-zone deep partial-thickness burn model in the mini-pig
Source: Burns Trauma. 2025 Feb 17;13:tkae086. doi: 10.1093/burnst/tkae086 (PMC11831022; doi:10.1093/burnst/tkae086)

**Figure S1 Tested brass pestle.**

The rectangular brass pestle measures 8x5x5cm and weighs 1.7 kg


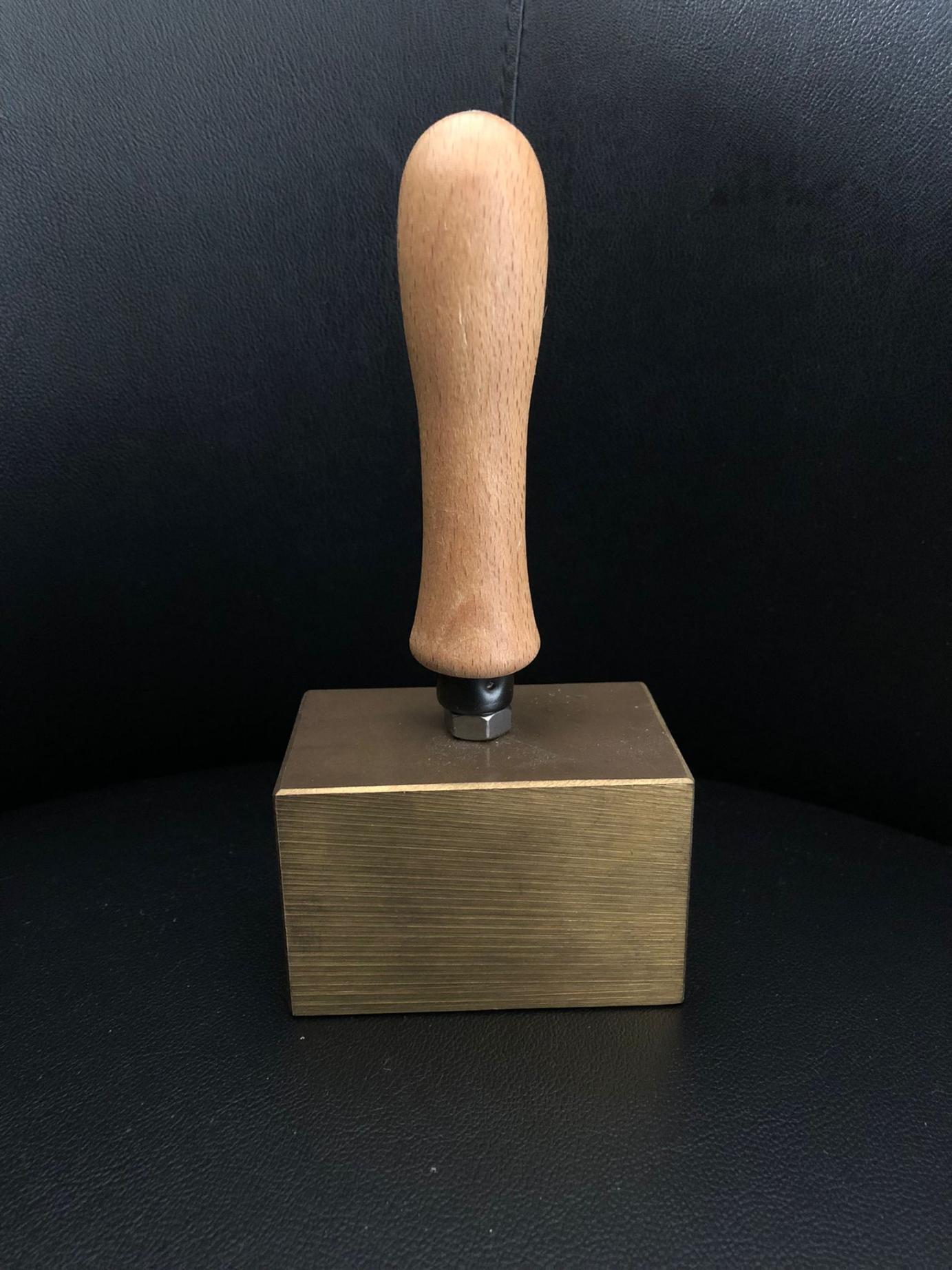


**Figure S2 Tested zones on the animals’ dorsal side (Study A)**

**
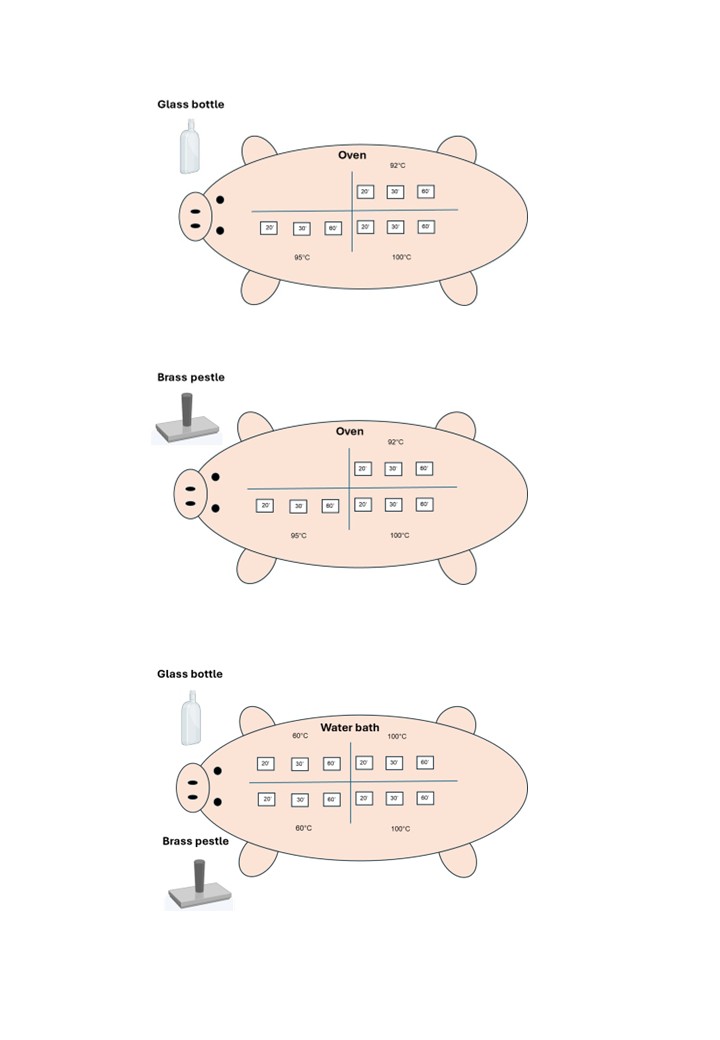
**

**Figure S3 Burns and biopsy sampling schema (Study B)**

**
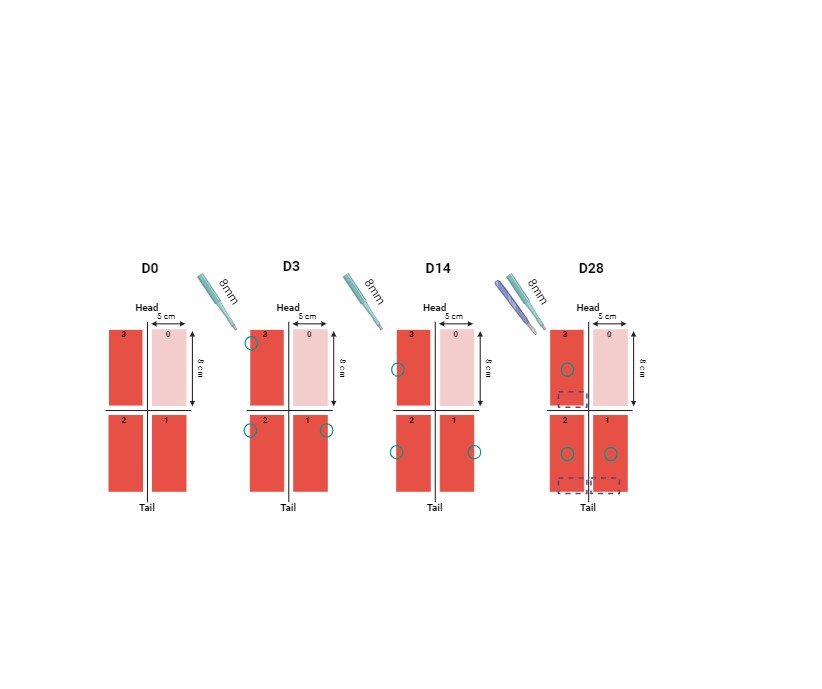
**

**Figure S4 Application of dressings (Study B).**

Dressings were changed every 3-4 days, for a total of 28 days, after cleaning the lesions with physiological solution


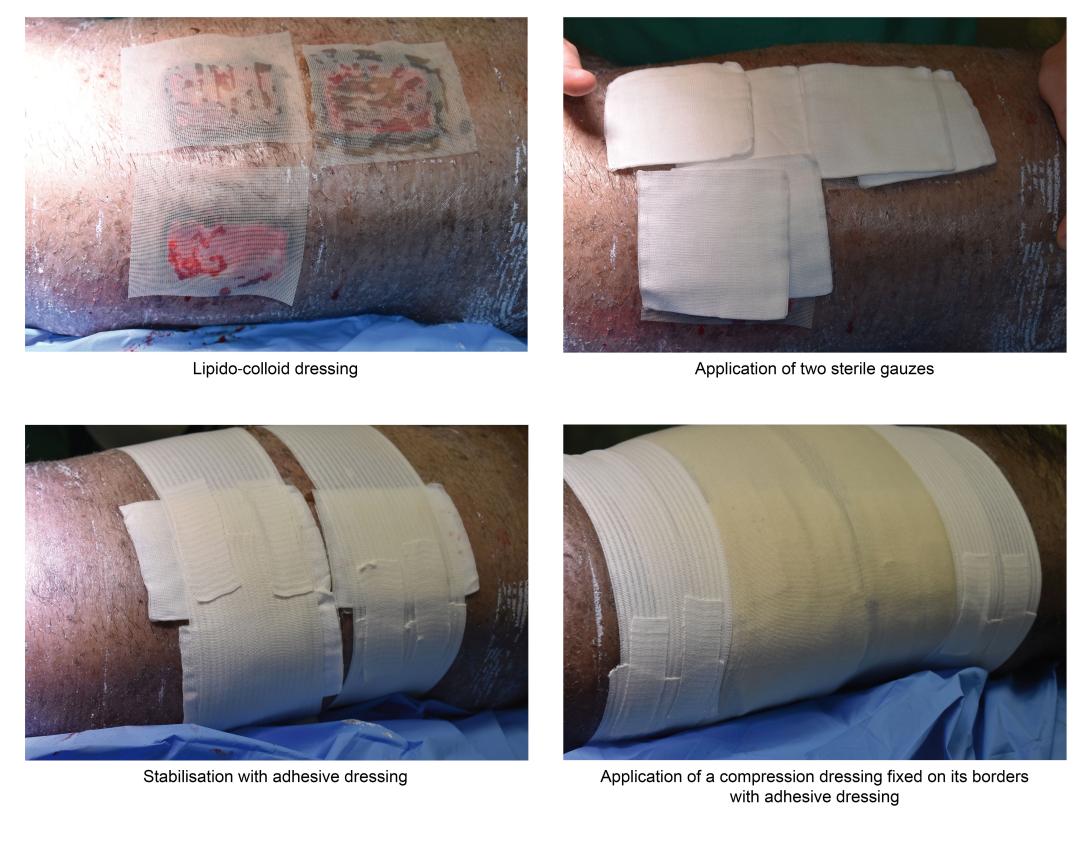


**Figure S5 Wound healing kinetics at D0 and at D28 (Study B).**

A 49% re-epithelialization of the lesion was observed after 28 days of post-burn care


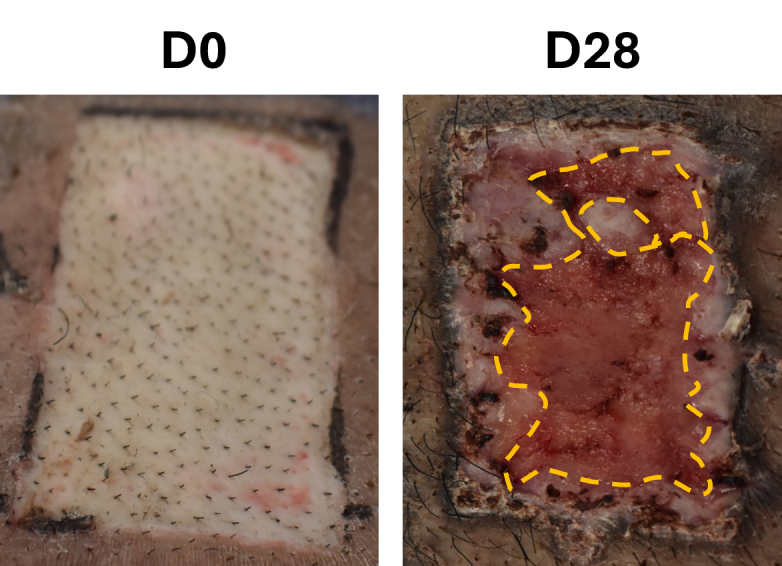

Supplement: Suppl_Figures_for_rev_05022025_tkae086 [file suppl_figures_for_rev_05022025_tkae086.docx]
